# The Global Anaerobic Regulator Anr, Is Involved in Cell Attachment and Aggregation Influencing the First Stages of Biofilm Development in *Pseudomonas extremaustralis*

**DOI:** 10.1371/journal.pone.0076685

**Published:** 2013-10-16

**Authors:** Paula M. Tribelli, Anthony G. Hay, Nancy I. López

**Affiliations:** 1 IQUIBICEN-CONICET and Dpto. de Química Biológica, Facultad de Ciencias Exactas y Naturales, Universidad de Buenos Aires, Buenos Aires, Argentina; 2 Department of Microbiology, Cornell University, Ithaca, New York, United States of America; University of Strathclyde, United Kingdom

## Abstract

*Pseudomonas extremaustralis* is a versatile Antarctic bacterium, able to grow under microaerobic and anaerobic conditions and is related to several non-pathogenic *Pseudomonads*. Here we report on the role of the global anaerobic regulator Anr, in the early steps of *P. extremaustralis* biofilm development. We found that the *anr* mutant was reduced in its ability to attach, to form aggregates and to display twitching motility but presented higher swimming motility than the wild type. In addition, microscopy revealed that the wild type biofilm contained more biomass and was thicker, but were less rough than that of the *anr* mutant. *In silico* analysis of the *P. extremaustralis* genome for Anr-like binding sites led to the identification of two biofilm-related genes as potential targets of this regulator. When measured using Quantitative Real Time PCR, we found that the *anr* mutant expressed lower levels of *pilG*, which encodes a component of Type IV pili and has been previously implicated in cellular adhesion. Levels of *morA*, involved in signal transduction and flagella development, were also lower in the mutant. Our data suggest that under low oxygen conditions, such as those encountered in biofilms, Anr differentially regulates aggregation and motility thus affecting the first stages of biofilm formation.

## Introduction

Biofilms, bacterial assemblies enclosed in a matrix, are found throughout many environmental biological niches. Cells forming these communities have advantages over their planktonic counterparts with respect to protection against both physical and chemical stresses [1]. Bacteria in biofilms are more resistant to antimicrobial agents and immune system surveillance [2], [3]. In addition, these structures provide protection against protozoa predation and environmental stresses such as cold and contaminants [4], [5].

The steps in biofilm formation have been studied extensively in *Pseudomonas aeruginosa* [6]. Several cellular functions such as motility, adhesion, metabolic switching, exopolysaccharides production and DNA and protein secretion are important during biofilm development. The expression of genes encoding these cellular functions is modulated during biofilm development [7], [8]. The role of global regulator proteins on biofilm formation has been studied in different species, for example, HhA and RpoS in *Escherichia coli*, CcpA in *Bacillus subtilis* and *Streptococcus mutans* and RpoN in *P. aeruginosa* [9], [10], [11], [12].

Biofilms are heterogeneous structures and microbial metabolism can vary dramatically depending on where cells are found within the biofilm [13]. Oxygen, in particular, can be in limiting supply within the biofilm and oxygen gradients have detected within biofilms. The mechanisms whereby cells sense and respond to oxygen are complex and not fully understood [14]. In *Pseudomonas*, the redox global regulator Anr controls anaerobic metabolism by activation and repression of targets genes. Denitrification, arginine and pyruvate fermentation, redox state maintenance, fimbria and cytochrome biosynthesis, secretion type III system, oxidative stress resistance and quorum sensing cascades are some of the functions that are known to be modulated by Anr [15], [16], [17], [18], [19], [20], [21], [22]. The role of global regulator Anr in biofilm development in non-pathogenic *Pseudomonas* species has not been studied yet.

*Pseudomonas extremaustralis,* a highly stress resistant bacterium isolated from Antarctic temporary water pond, is able to synthesize high amounts of poly(3-hydroxybutyrate) (PHB) [23], [24]. PHB production is important for the transition between biofilm and planktonic lifestyles under cold conditions [5].Biofilms increase diesel degradation in *P. extremaustralis* [25]. Under microaerobic or anaerobic conditions, *P. extremaustralis* reduces nitrate but it is unable to perform complete denitrification because it lacks nitrite reductase genes [26], [27].

In this work we analyzed the role of Anr in the early steps of biofilm formation by *P. extremaustralis.*

## Materials and Methods

### Bacterial Strains and Growth Conditions

*Pseudomonas extremaustralis* DSM 25547 and an *anr* mutant containing a 250-bp deletion and a kanamycin cassette insertion were used throughout this study [26], [27]. A complemented strain was constructed as described before, by inserting the entire wild type *anr* sequence in the mutant by using a mini-Tn5 delivery system [21]. All cultures were performed using 0.5 NE2 medium supplemented with 0.2% glucose, 0.3%, casaminoacids, 0.08% KNO_3_, 1 mM MgSO_4_ and 0.1% micronutrients [28]. Microaerobic cultures were performed in 100-ml hermetically sealed bottles containing 50 ml of culture medium. Bottles were incubated at 30°C and low shaking (75 rpm) to avoid cellular aggregation.

### Biofilm Experiments

Biofilms were grown in glass bottom Petri dishes (Mat Tek, 3 mm Petri-dish, 14 mm microwell and 1.0 mm coverglass) with 3 ml of 0.5 NE2 supplemented medium as described above with slow shaking (50 rpm). The medium was inoculated with overnight cultures to give an initial OD_600_ of 0.025. Culture medium was replaced every 24 h and the OD_600_ and the CFU/ml of planktonic cells were determined. For further microscopy visualization of the biofilm, 3 ml of low melting point agarose (1%) was added as the culture medium was withdrawn in order to maintain biofilm structure. The experiment was performed by triplicate.

### Attachment Experiments

Initial attachment was studied by using 96-well polystyrene microtiter plates (Gibco), as described by O’Toole and Kolter [29]. Briefly, 200 µl of each culture were added to the microplate wells and incubated at 30°C without agitation for 3 h. Non-attached cells were collected and OD_600_ was measured (absorbance of planktonic cells: APL). Biofilm attached cells were stained with 200 µl 0.1% crystal violet. After 20 min, the unbound crystal violet solution was removed and plates were gently rinsed with water. Subsequently, the crystal violet was extracted from the bound cell with 200 µl 96% ethanol for 20 min and transferred to flat bottom microtiter plates in order to measure the absorbance at 550 nm (absorbance of crystal violet: ACV) in a Tasoh Corp MPR A4i microplate reader. The attachment index was defined as ACV/APL.

### Autoaggregation Assays

Autoaggregation and settling assays were performed as described Sherlock et al. [30] with modifications. Briefly, overnight microaerobic cultures were diluted with fresh media and incubated until 0.8 OD_600_. A 1 ml aliquot was incubated at room temperature without agitation for 3 h and stained with 4′,6-diamidino-2-phenylindole (DAPI) for fluoresce microscopy or with a 4% aqueous solution of uranyl acetate for electronic microscopy. After that, 200 µl from the top 5 mm of the culture was taken (non- settled) while the rest of the culture was vigorously vortexed and the OD_600_ of both samples was determined. Aggregation % was determined as follows: (OD vortexed-OD non-settled)/OD vortexed × 100.

### Microscopy

For DAPI visualization an Olympus BX40 microscope with a UV lamp was used. For transmission electron microscopy (TEM) samples were allowed to adhere to carbon-coated 200 mesh grids and were stained with uranyl acetate. TEM was performed with a Philips EM 201 microscope. Twitching motility was visualized under a Leica DFC300X microscope using contrast phase mode using 400× magnification.

### Motility Experiment

Swimming motility was evaluated using a plate assay [31]. 5 µl of an overnight culture was used to inoculate swimming medium plates containing 10 g/l bacto-tryptone, 5 g/l NaCl, 0.3% wt/vol agarose, 0.3% casaminoacids and 0.2% glucose. Swimming distance was measured after 24 h. Twitching motility was visualized using the slide culture method [32]. Briefly, microaerobic cultures were used to point inoculate onto the surface of a LB agar (1%) slice placed on a microscope slide. The inoculum was covered with a coverslip and incubated at 30°C for 15 h in a humid environment. The samples were visualized by contrast phase microscopy.

### Quantitative Real Time PCR Experiments

Total RNA was extracted from 24 h old microaerophilic cultures of *P. extremaustralis* and the *anr* mutant using the RNeasy Mini kit (Qiagen). After treatment with DNaseI, cDNA was obtained using random hexamers (Promega) and AMV retrotranscriptase following the manufacturer’s instructions. At least three independent cultures were analyzed for each strain. RT-qPCR was performed using a LightCycler (DNA Engine M.J. Research) and Real Time PCR mix (Biocientist, no Rox). Three genes were analyzed using the following primers: *pilG* 5′TCCCGGTGATCATGCTGTCCTCC3′ and 5′TGTTCTACTGCCGCGAACCCA3′; *morA* 5′GGTTGCGGGACAACCCCATCG3′ and 5′ GGTGGTGTTACGCGGGCAGTC3′ and 16S rRNA gene 5′AGCTTGCTCCTTGATTCAGC3′ and 5′AAGGGCCATGATGACTTGAC3′ employed as reference for normalization of expression levels of target genes in each strain. The thermocycler conditions were as follows: denaturation at 95°C for 5 min; 40 cycles at 95°C for 25 s, 52.3°C for 15 s, and 72°C for 15 s; with fluorescence acquisition at 80°C in single mode. Relative changes in the expression of individual genes in both strains were obtained through the relative standard curve method [33].

### Biofilm Visualization

*P. extremaustralis* wild type and the *anr* mutant strain were transformed with plasmids encoding GFP in order to analyze biofilm structure [34]. Biofilms were visualized using an Olympus BX61 microscope equipped with a 620 low-power objective with WG and FITC fluorescence filter cubes (Olympus). Samples were illuminated using a Lambda LS Xenon arc lamp (Sutter Instruments). Images were acquired using a Cooke SensiCam with a Sony Interline chip. The image capture size was 512 x 512 pixels and the Z section step size was 1 µm. Image acquisition, nearest neighbor deconvolution and 2D image production were performed using the SlideBook Software package version 3.0.10.15 (Intelligent Imaging). At least six stacks for each sample were analyzed with COMSTAT2 software [35] available on line using the automatic threshold option.

### Bioinformatic Analysis

The complete genome sequence of *P. extremaustralis* has been deposited at DDBJ/EMBL/GenBank under the accession no. AHIP01000001.1- AHIP01000135.1 [27].The Anr regulon of *P. extremaustralis* was predicted using the Virtual Footprint tool available in PRODORIC software [36]. Putative target genes were considered only when the Anr-box was located within 300 bp upstream from the start ATG codon, based on previous data with experimental support [37], and in an intergenic genomic zone. Putative σ^70^ dependent promoters were identified using the Softberry Bprom algorithm (http://linux1.softberry.com/berry.phtml). Sequence logos was performed using 5 Anr-boxes belonging to *morA* and *pilG* genes and also those found in previous works to be influenced by Anr in *P.extremaustralis anr* strain in previous work [26], [27].

### Statistics Tests

The significance of each treatment was evaluated by the Student’s t test with P<0.05 considered as significant. In attachment experiments the treatments between strains and time were evaluated by ANOVA test.

## Results

### Biofilm Formation is Influenced by Anr

The wild type formed well-defined microcolonies by 24 h (data not shown) and had the highest value for total biomass and average thickness after 72 h, though the roughness coefficient was lowest at that time point (Table 1). At 96 h a decrease in total biomass and thickness was observed probably due to a dispersion phenomenon. In contrast, biofilms of the *anr* mutant strain had significantly lower total biomass as well as average and maximum thickness at all the assayed times (Table 1). The R value was higher, however, for the mutant strain biofilms than for the wild type biofilms, suggesting a more disorganized structure (Table 1). Due to the defects observed in biofilm formation in the mutant strain and the importance of Anr in anaerobic metabolism, we investigated the culturability of planktonic cells. There was not statistical significance in the numbers of culturable planktonic cells for the time points except at 96 h, when the *anr* strain numbers (2.10±5.10 CFU ml^−1^) exceeded those of the wild type (6.10±2.10 CFU ml^−1^) (P<0.05).

**Table 1:** Biofilm parameters of wild type and *anr* mutant *of P. extremaustralis*. Values represent the mean ± SD of three independent experiments.

Parameters	strain	24 h	48 h	72 h	96 h
Biomass (Bio-volume, µm^3^. µm^−2^)	Wild type	17.2±4.4	13.9±3.2	23.2±4.6	14.2±2.5
	*anr* strain	2.2±0.3*	6.1±2.6*	0.53±0.5*	1.2±0.8*
Average thickness (µm)	Wild type	22.5±7.2	22.3±6.9	34.4±5.5	18.3±3.4
	*anr* strain	2.5±0.8*	14.7±5.9	1.7±1.5*	2.6±2.0*
Maximum thickness (µm)	Wild type	100.0±26.3	92.0±13.0	81.6±11.6	68.4±20.0
	*anr* strain	60.5±10.2*	63.7±20.0	65.2±12.7*	70.0±11.6
Roughness coefficient	Wild type	1.4±0.1	1.3±0.3	0.9±0.2	1.1±0.2
	*anr* strain	1.6±0.4	1.6±0.2	1.7±0.4*	1.6±0.3*

The asterisk (*) denotes significant differences among strains (P<0.05) using the Student’s t test.

### Anr Enhances Attachment, Autoaggregation and Twitching Motility and Decrease Swimming Motility

**Figure 1:**
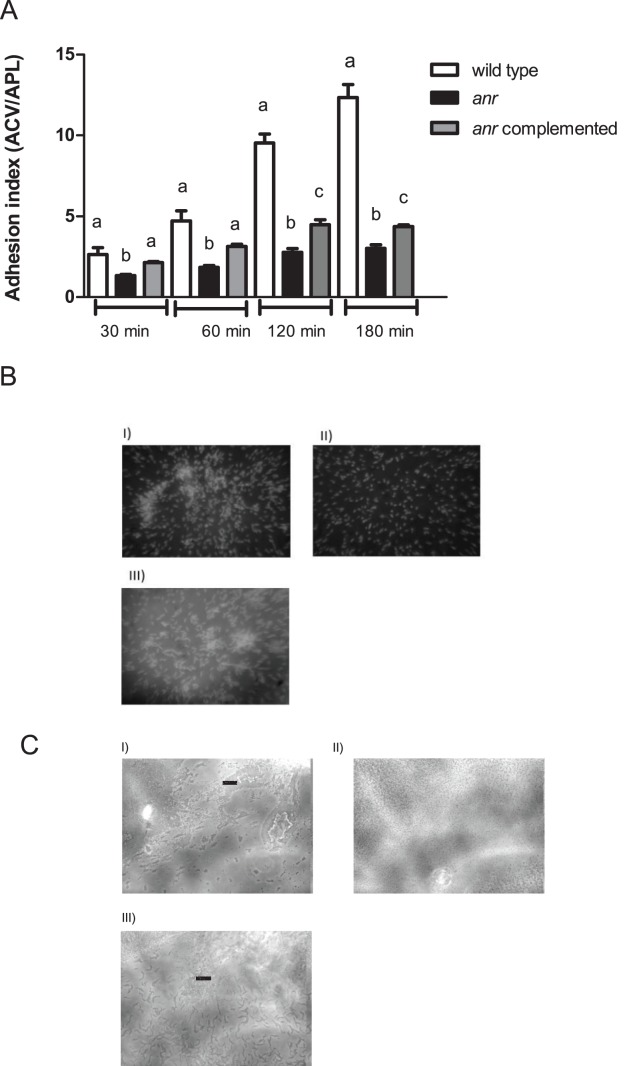
Anr absences decrease attachment and aggregation in *P. extremaustralis*. (A) Attachment to polystyrene plates in 0.5 NE2 medium supplemented with glucose, KNO_3_ and casaminoacids. Values represent media ± SD of 5 independent experiments with 12 wells per strain. Different letters showed significant differences among strains (P<0.05) using ANOVA (B). Autoaggregation experiment. The cells were incubated during 2 h without agitation and a culture sample was stained with DAPI. I) wild type strain. II) *anr* mutant strain. III) *anr* complemented strain. All observations were performed at 1000X. (C). Slide culture assay to investigate twitching motility. Cells were incubated for 15 h. I) wild type strain. II) *anr* mutant strain. III) *anr* complemented strain. Arrows showed rafts in the edge of the culture. All observations were performed using contrast phase microscopy at 400× magnification.

Biofilm development includes different stages, among them; adhesion to surface and cellular aggregation constitute important earlier steps. The wild type strain incremented significantly the adhesion index throughout the experiment (P<0.05) while the *anr* mutant strain showed a slight increment of this parameter with only significantly differences in the first assayed times. The values of the adherence index were significantly higher in the wild type strain in all times in comparison with the *anr* mutant strain (Fig. 1A). The complemented strain presented a partial restoration of this phenotype (Fig. 1A).

Additionally, settling capability, which is a common measure of cell to cell adhesion, was significantly higher in the wild type than the *anr* mutant, with values of 48±13% of aggregation for the wild type and 17±5% for the *anr* (P<0.05). This aggregation defect was partially restored by complementation (33±6%). Aggregation by the complemented was significantly different than the *anr* strain but not significantly different than the wild type (P<0.05 and P>0.05, respectively). Microscopic observation of DAPI stained cells showed that the wild type and the complemented strain aggregated in clumps while mostly single cells were observed in the *anr* mutant culture (Fig. 1B). Another important feature in biofilm formation is twitching motility. We assayed the twitching capability in cells belonging to microaerobic cultures. The examination of the wild type slide culture revealed the presence of motile rafts of cells at the leading edge of the moving zone while in the mutant strain; the cells were dispersed without forming rafts (Fig. 1C). The complemented strain presented a pattern similar to that observed in the wild type strain (Fig. 1C).

**Figure 2:**
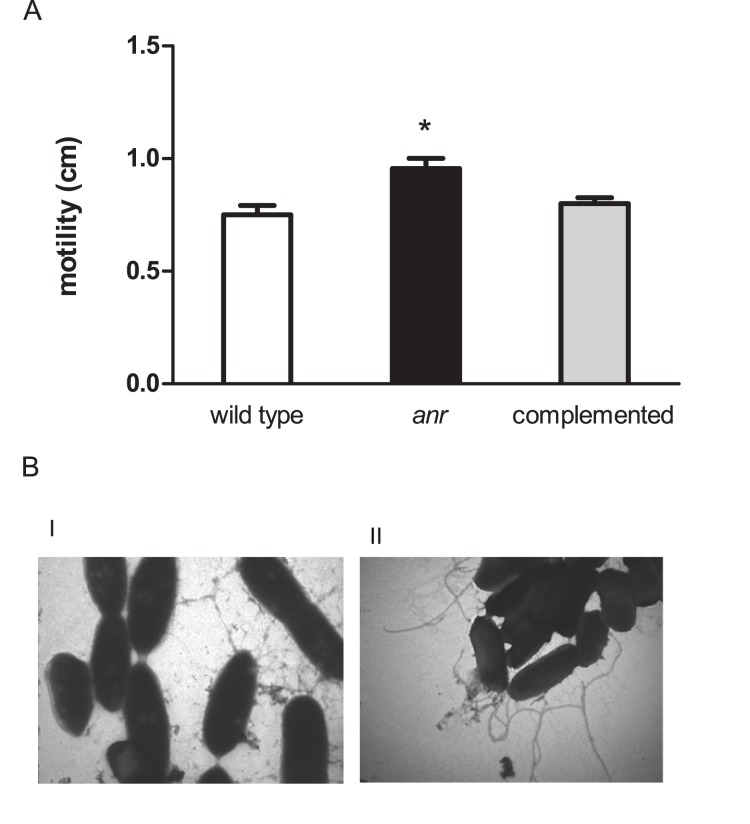
Swimming motility is increased in *anr* mutant strain. (A) Swimming motility. The asterisk (*) denotes significant differences (P<0.05) using the Student’s t test. (B) Transmission electron microscopy. I) wild type strain. II) *anr* mutant strain. Observations were performed at 46000X magnification.

The mutant cells were also visibly more motile than the wild type when viewed under the microscope (data not shown). Given this observation and the importance of motility in the early stages of biofilm development, swimming motility was also evaluated in motility agar. As expected, the wild type and the complemented strain were significantly less motile than the *anr* mutant strain (Fig. 2A). Consistent with these results, flagellar-like structures were only visible in TEMs of the *anr* mutant and not in those of the wild type (Fig. 2B).

### Anr Controls the Expression of Genes Involved in Biofilm Development

**Figure 3:**
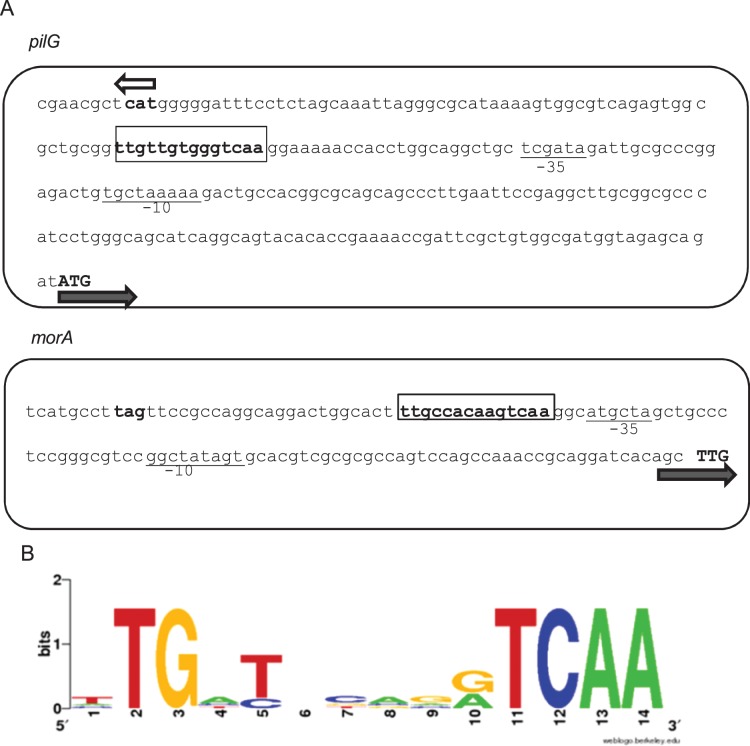
Organization of the P. *extremaustralis* intergenic *pilG* and *morA* region showing putative Anr boxes. (A) The sequences −35 and −10 of a probable σ^70^ promoter are underlined. The *pilG* and *morA* start codons and the start or the stop codon of the neighbors genes are shown by boldface type and arrows indicate the direction of the transcription. Anr boxes are boxed. (B) Sequence logo of 5 Anr boxes located in genes influenced by Anr in *P. extremaustralis* were used to generate the Anr position weight matrix sequence.

**Figure 4:**
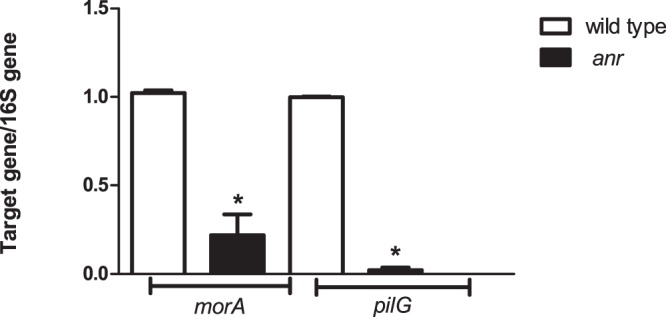
Effect of *anr* mutation on expression of *pilG* and *morA* genes in microaerobic cultures. (A) qPCR Real Time experiments were carried out in microaerobic cultures. Values were expressed as the ratio between the raw level of expression of each target gene and the 16S rRNA gene, and represent the mean ± SD of three independent experiments. The asterisk (*) denotes significant differences (P<0.05) using the Student’s t test.

The *P. extremaustralis* genome sequence was analyzed to determine putative targets genes for Anr regulation. Two target genes involved in different biofilm functions were detected in which the Anr-box was located in an intergenic zone, *pilG* and *morA* (Fig. 3). *pilG*, is relevant to Type IV pili assembly in other *Pseudomonads* whereas *morA* encodes a repressor of flagella development [38], [39]. The logos performed with the Anr-boxes located in the putative promoter zone of *pilG* and *morA* and also the Anr-boxes *of phaBAC, cioA and cooN* of *P. extremaustralis* showed a similar *P.aeruginosa* Anr-box consensus sequence [37]. We performed Quantitative Real Time PCR experiments to determine if expression of *pilG* and *morA* was altered in the *anr* mutant strain. The expression of both genes in the mutant strain was lower than the wild type (Fig. 4A). The lower pilG expression is in line with the defects on twitching, adhesion and aggregation in the *anr* mutant strain. The *morA* altered expression was consistent with the higher swimming motility observed in absence of *anr.*

## Discussion

Biofilms development constitutes a complex process influenced by a variety of factors including oxygen availability [13]. In *Pseudomonas* species Anr controls several components of the response to low oxygen availability. In *P. aeruginosa*, the best studied member of the genus, Anr transcriptional levels were found to be up-regulated in confluent biofilms and it has been demonstrated that Anr controls quorum sensing by regulating the expression of the small regulatory RNA PhrS [14], [22].Recently, Jackson et al. [40] observed that Anr was crucial for *P. aeruginosa* virulence in a mouse model and that biofilm formation was defective in an *anr* mutant, but the mechanism behind this deficit was not described. The importance of global regulators that affect biofilm formation has also demonstrated with *arcA* in *Escherichia coli* [41]. These reports suggest a relationship between oxygen availability and physiological responses mediated by quorum sensing and biofilm development.

In this work, microscopic analysis showed that an *anr* mutant of *P. extremaustralis* had impaired biofilm development. This was not simply due to a growth deficit, since the number of culturable planktonic *anr* cells was the same or greater than the number of wild type cells. The reduction in biofilm formation can be attributed to two specific phenotypic defects that are known to be important in the early steps of biofilm formation, namely, the *anr* mutant strain showed lack of cellular aggregation and was highly motile. Newell et al. [42] have shown that biofilm initiation is result of a combined reduction of motility and an increase in adhesion. Thus, both flagella and Type IV pili are important to biofilm development in glucose supplemented cultures [43]. Pili serve to assist in attachment of cells to surfaces and twitching motility whereas flagella have a dual function since are important for attachment but are also involved in dispersion [44]. In the present work, we demonstrated that Anr is involved in aggregation and motility in *P. extremaustralis*, since an *anr* mutant was deficient in the attachment to polystyrene plates, autoaggregation, and twitching motility while presented a higher swimming motility which is dependent of flagella.

Several genes encode functions that contribute to biofilm development, including those encoding regulators or components of surface structures such as pili and flagella including, for example *pilG, pilA*, *fliC* [44], [45]. Our *in silico* analysis revealed that several *P. extremaustralis*, genes relevant to these structures had putative Anr boxes including *pilG* and *morA*. Quantitative real time PCR confirmed that both these genes were down regulated in the *anr* mutant. The reduction of *pilG* expression levels in the *P. extremaustralis anr* mutant likely impaired pili biosynthesis and resulted in the aggregation and twitching defects we observed. Something similar has been observed in *P. aeruginosa* Crc mutants where the mutations in this regulatory protein lead to pili defects and concomitant defects in biofilm formation [46].

Additionally, we found that transcripts of *morA*, predicted to encode a protein similar to those involved in signal transduction, were less abundant in the *P. extremaustralis anr* mutant. Studies of *morA* mutants of *P. putida* showed increased motility and an inability to develop biofilms [39] which is similar to our observations of the *P. extremaustralis anr* mutant in this work. Interestingly, in *P. aeruginosa morA* mutant strains neither motility nor biofilm formation are affected, suggesting a different mechanism in this species [39].

Taken together, the results presented here demonstrate that Anr is involved in regulating the autoaggregation, adhesion and twitching and swimming motility of *P. extremaustralis* and that the loss of Anr impairs biofilm development. Although different signals also regulate aggregation and motility [47], [48], [49] the importance of Anr in biofilm development indicate oxygen availability as a signal that regulates biofilm development in *P. extremaustralis.*
